# Protein phosphatases in TLR signaling

**DOI:** 10.1186/s12964-021-00722-1

**Published:** 2021-04-21

**Authors:** Clovis H. T. Seumen, Tanja M. Grimm, Christof R. Hauck

**Affiliations:** 1 Lehrstuhl Zellbiologie, Universität Konstanz, Universitätsstraße 10, Postablage 621, 78457 Konstanz, Germany; 2 Konstanz Research School Chemical Biology, Universität Konstanz, 78457 Konstanz, Germany

**Keywords:** Protein serine/threonine phosphatase, Toll-like receptors, Innate immunity, Phosphorylation, Inflammation, NF-κB (nuclear factor kappa-light-chain-enhancer of activated B cells)

## Abstract

Toll-like receptors (TLRs) are critical sensors for the detection of potentially harmful microbes. They are instrumental in initiating innate and adaptive immune responses against pathogenic organisms. However, exaggerated activation of TLR receptor signaling can also be responsible for the onset of autoimmune and inflammatory diseases. While positive regulators of TLR signaling, such as protein serine/threonine kinases, have been studied intensively, only little is known about phosphatases, which counterbalance and limit TLR signaling. In this review, we summarize protein phosphorylation events and their roles in the TLR pathway and highlight the involvement of protein phosphatases as negative regulators at specific steps along the TLR-initiated signaling cascade. Then, we focus on individual phosphatase families, specify the function of individual enzymes in TLR signaling in more detail and give perspectives for future research. A better understanding of phosphatase-mediated regulation of TLR signaling could provide novel access points to mitigate excessive immune activation and to modulate innate immune signaling.

**Video Abstract**

**Supplementary Information:**

The online version contains supplementary material available at 10.1186/s12964-021-00722-1.

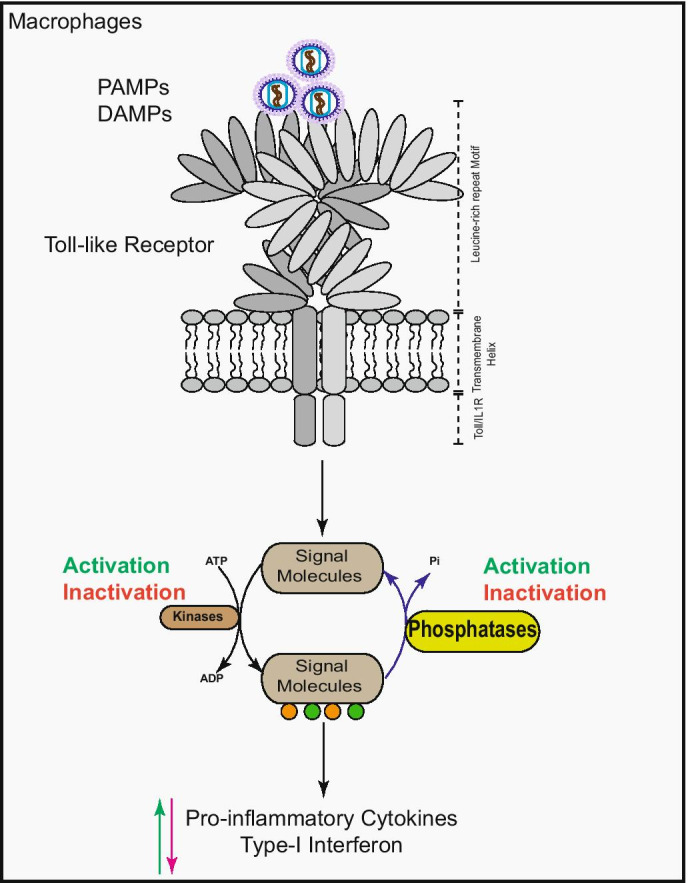

## Background

Toll like receptors (TLRs) sense pathogen-associated molecular patterns (PAMPs) or damage-associated molecular patterns (DAMPs) by innate immune cells, but also by various other cell types [1]. Upon ligand binding, TLRs trigger downstream signaling cascades, which impinge on the activation of transcription factors such as activation protein-1 (AP-1), interferon regulatory factors (IRFs), and nuclear factor kappa-light chain enhancer of activated B-cells (NF-κB). The latter is a major driver of the production of type I interferon and pro-inflammatory cytokines [2]. Consequently, the initiation of TLR signaling triggers inflammation and activates innate and adaptive immune responses [3–5]. As the excessive activation of this pathway can lead to life-threatening conditions, TLR signaling has to be tightly regulated [6]. In fact, an elaborated regulatory network controls TLR signaling: while activation relies on protein phosphorylation, ubiquitination, and selective protein–protein interactions, these processes are counterbalanced by the activity of protein phosphatases and deubiquitinating enzymes. The last decades have witnessed a dramatic increase in our understanding of different protein serine/threonine kinases and their target molecules in TLR signaling [7, 8]. However, we have still limited knowledge of specific protein serine/threonine phosphatases (PSPs) involved in counteracting TLR-initiated kinase signaling [9]. Given the widespread occurrence and important role of protein phosphorylation in TLR signaling, it is apparent that numerous PSPs participate in the regulation of these events. Intriguingly, the identities of most of these enzymes and their particular substrates still await clarification. In this review, we want to summarize the current knowledge about PSPs and their role in TLR signaling. Following an overview of phosphorylation events along the TLR signaling cascade, we will highlight the involved kinases and phosphatases. In the second part, we provide a more detailed description of individual PSP families, to identify gaps in our knowledge and suggest directions for future research in this area.

## Part I: the role of protein phosphorylation in TLR signaling

Balancing phosphorylation and dephosphorylation of proteins is pivotal to initiate, drive, and terminate TLR signaling. Right from the initial events at the plasma membrane, cytoplasmic protein kinases with an activity directed against serine or threonine residues in their substrates (Ser/Thr protein kinases) are key drivers of TLR signaling. Representatives of this group of enzymes can be found in complex with the receptor at the plasma membrane, in a large, multi-component signalosome in the cytosol, and as regulators of gene expression in the nucleus. In fact, three main Ser/Thr protein kinase families are conserved elements of TLR signaling: Interleukin-1 receptor-associated kinases (IRAKs), the transforming growth factor-β-activated kinase 1 (TAK-1), and the IκB kinase (IKK) complex. However, additional serine/threonine kinases such as NIK, MST4, TBK-1, TANK, and RIPK also contribute to TLR-initiated processes [6, 7, 10–12]

IRAK family members (IRAK1, IRAK2, IRAKM, IRAK4) play a central role in TLR/IL-1 signaling. Upon ligand binding to the TLR and association with the adapter molecule MyD88, IRAKs are promptly recruited to MyD88 through death-domain interactions [13]. IRAK4 is a decisive kinase in the TIR signaling pathway and is the first enzyme to be recruited to the Myddosome complex by MyD88/TIRAP or TRIF/TRAM. Human patients with inherited IRAK-4 deficiency as well as IRAK4 knock-out mice exhibit severe functional defects in TLR pathways [14]. In line with these in vivo findings, cells lacking IRAK4 show impaired TLR pathway responses [15, 16]. Clustering of IRAK4 together with MyD88-bound TLRs results in dimerization of the kinase and trans-phosphorylation of residues within the kinase domain. Therefore, mutations in IRAK4 that abrogate IRAK4 kinase activity (IRAK4 G298D) or disrupt the interaction with MyD88 and IRAK1 (IRAK4 R12C) lead to reduced IL-1-induced signaling and cytokine production [17]. Upon activation, IRAK4 recruits IRAK1 (earlier phase) and IRAK2 (late phase) as well as TRAF6 and Pellino to the Myddosome complex [16, 18–20]. Upon binding, IRAK1 is phosphorylated by IRAK4 on a key threonine residue (Thr209) in the kinase domain. This phosphorylation triggers conformational changes in IRAK1, which facilitate autophosphorylation in the kinase activation loop (Thr387) as well as hyperphosphorylation in the so-called proline, serine and threonine (ProST) region of IRAK1. Once activated, IRAK1 is released from the Myddosome, but remains associated with TRAF6, an E3 ubiquitin ligase. IRAK1, but also IRAK2 and IRAK4, can phosphorylate TRAF6 and the E3 ubiquitin ligase Pellino on several residues [21, 22]. For instance, Pellino 1 has been reported to be phosphorylated on seven residues within the forkhead-associated domain and two residues within the RING-like domain [23]. Activation of the E3 ubiquitin ligases Pellino and TRAF6 via phosphorylation and their concomitant K63-linked poly-ubiquitination promotes the recruitment of additional Ser/Thr kinases [24]. At the same time, TRAF6 and Pellino act back on their upstream kinase, IRAK1, to induce the poly-ubiquitination and proteasomal degradation of this enzyme, thereby creating a negative feedback loop [25].

Signal propagation by poly-ubiquitinated TRAF6 occurs through association with the Transforming Growth Factor-β-activated kinase 1 (TAK1) in complex with TAK1 binding protein-1/2 (TAB1/2). The TAK1/TAB complex is activated by binding to K63 poly-ubiquitin chains, such as those attached to TRAF6, and by phosphorylation at Thr184, Thr187 and Ser412 in the TAK1 kinase domain [26–28]. Active TAK-1 in turn phosphorylates the IκB kinase β (IKKβ) at key serine residues within the activation loop at Ser176 and Ser180 [8]. IKKβ resides together with a closely related kinase, IKKα, and the scaffold protein IKKγ (also termed NEMO) in a cytosolic complex [8]. Upon stimulation by TAK1, the activity of IKKβ is directed towards the Inhibitor of NF-κB (IκBα), which is phosphorylated by active IKK on serine residues Ser32 and Ser36 [29]. Phosphorylation at these amino-terminal serine residues marks IκBα for poly-ubiquitination via Lys48-connected poly-ubiquitin chains, which initiate the efficient proteasomal degradation of this NF-κB inhibitor. Once the NF-κB heterodimer (p50/p65) is released from its protein inhibitor IκBα, the nuclear localization signal within NFκB is accessible and the transcription factor can translocate to the nucleus. There, NF-κB stimulates the transcription of a large set of genes via binding to characteristic kB sites located in the promoter regions of target genes [30, 31].

**Fig. 1:**
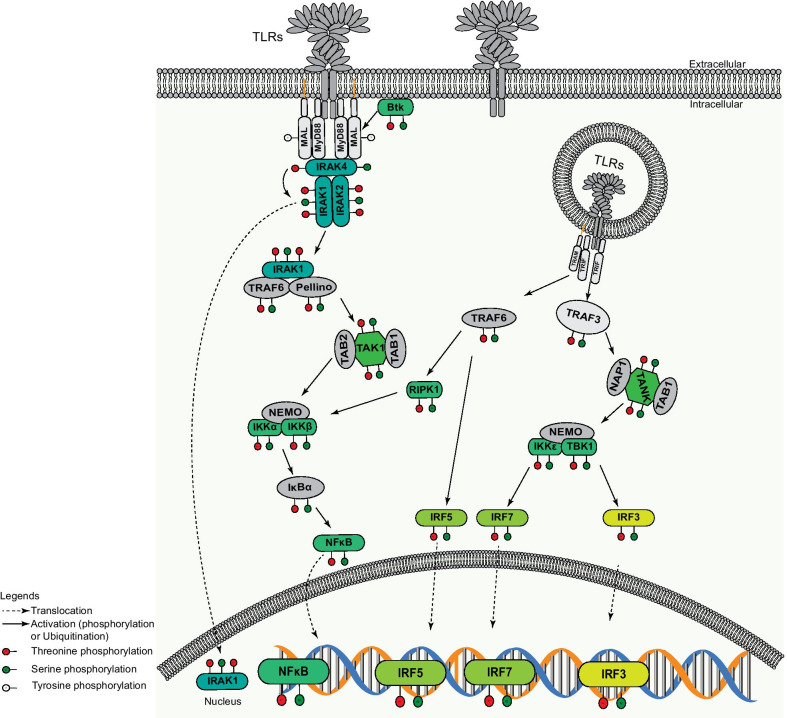
Phosphorylation events in TLR signaling. TLRs recognize their respective ligands at the cell surface or endosomes, leading to recruitment of MyD88. A receptor complex (Myddosome or Triffosome) is formed with IRAK4 and IRAK1 (and IRAK2 in the late phase). IRAK4 phosphorylates IRAK1, and the TRAF6-IRAK1 complex dissociates from MyD88. IRAK1 undergoes autophosphorylation and is degraded by the proteasome upon Pellino-1-mediated polyubiquitination. Pellino-1 and TRAF6 recruit TAK1-TAB2-TAB1 complex and IKKα/β-NEMO Complex. TAK1 is autophosphorylated and phosphorylates IKKα/β-NEMO Complex. Subsequently, IKKα/β phosphorylates IκBα bound to NF-κB p65. The latter is released and translocates to the nucleus. In the TRIF dependent pathway, TRAF6 recruits either RIPK1 and activates the IKK-IκBα-NF-κB p65 axis, or IRF5. On the other hand, TRAF3 recruits NAP1-TANK-TAB1 and NEMO-IKKε-TBK1 complexes, upon which TANK phosphorylates and activates IKKε and TBK1. Both kinases trigger IRF3 or IRF7-mediated type-I interferon production. Phosphorylated tyrosine, serine and threonine residues are represented by white, green, and circles red, respectively. Activation (ubiquitination or phosphorylation) and translocation are indicated by plain or dashed arrows, respectively

Already this short summary of canonical TLR-initiated processes illustrates the multitude of phosphorylation events, which govern this particular signaling pathway inside cells and which are summarized in Fig. 1. It is also evident from this description that Ser/Thr kinases are main actors in TLR signaling. Clearly, these kinase-mediated phosphorylation events have to be counterbalanced by serine-threonine-directed protein phosphatases to control the magnitude and duration of pathway output. However, in contrast to the detailed knowledge we have about the involved kinases, only little is known about protein phosphatases, which are directed against particular substrate proteins and specific phospho-residues in the TLR signaling cascade. This lack of knowledge is even more striking given that the human genome contains only a limited set of serine/threonine-directed protein phosphatases (see Part II of this review), which contrasts the > 400 distinct serine-threonine kinases found in mammals [32, 33]. Some of the known dephosphorylation events concern IRAK1, the kinase embedded within the Myddosome [20]. IRAK1 has been co-immunoprecipitated together with the serine/threonine phosphatase PP2A and treatment with okadaic acid, a potent inhibitor of PP2A activity, or siRNA-mediated depletion of the catalytic subunit of PP2A results in increased ubiquitination and degradation of IRAK1 in response to IL1β stimulation [34]. These findings suggest that the PP2A-regulated phospho-sites on IRAK1 are involved in orchestrating IRAK1 poly-ubiquitination and turnover by the proteasome. This would imply that PP2A activity stabilizes IRAK1 and sustains TLR signaling, while diminishing PP2A activity should negatively impact TLR signaling. On the contrary, PP1 and PP2A have been shown to completely dephosphorylate IRAK1 in vitro suggesting that they can act on several phospho-sites within this enzyme [35, 36]. As the phosphorylation state of particular residues can either have positive effects on IRAK1 activity (e.g. due to phosphorylation of IRAK1 residue Thr-387 within the kinase activation loop) or negative consequences (e.g. due to phosphorylation-dependent poly-ubiquitination leading to proteasomal degradation) it is instrumental to clearly define the particular site(s) of action of a given protein phosphatase to understand the consequences of this enzyme’s activity on TLR signaling output. Interestingly, the individual IRAK1 phospho-residues targeted by either PP2A or PP1 and the kinetics of their activity have not been elucidated so far [37]. This appears to be true for most of the various protein phosphatases, which have been implied in the TLR signaling pathway. Besides PP1 and PP2AC, the phosphatase enzymes known to affect TLR signaling are PP2B, PP4, PP6, PPM1A, PPM1B, PPM1D, PPM1E, PPM1F, PPM1L, and PPM1M. In the second part of this review, we will address the individual phosphatases and detail their known points of action along the TLR signaling cascade, but also highlight current shortcomings in our knowledge.

## Part II: protein serine/threonine phosphatases in TLRs signaling

Negative regulation of TLR signaling is mediated by several mechanisms. Besides inhibition of kinase activity and poly-ubiquitin-initiated protein degradation, the dephosphorylation by protein phosphatases is a rapid and reversible means to limit TLR pathway output. Therefore, there is growing interest in understanding the role of different protein phosphatases in TLR signaling. Protein serine/threonine phosphatases (PSPs) in general are subdivided into three families based on structural homology: phospho-protein phosphatases (PPPs; with 13 PPPs encoded in the human genome), metal-dependent protein phosphatases (PPMs; with 16 PPMs found in humans) and aspartate-based phosphatases (including FCP/SCP- and HAD-type enzymes; with 21 members) [33, 38, 39]. As protein phosphorylation is one of the major posttranslational modifications, protein phosphatases are involved in every physiological process including cell division, cell proliferation and differentiation, programmed cell death as well as the regulation of the immune response. Here, we will focus on those family members with a known direct contribution to TLR signaling.

## PPP family members in TLR pathway regulation

**Fig. 2:**
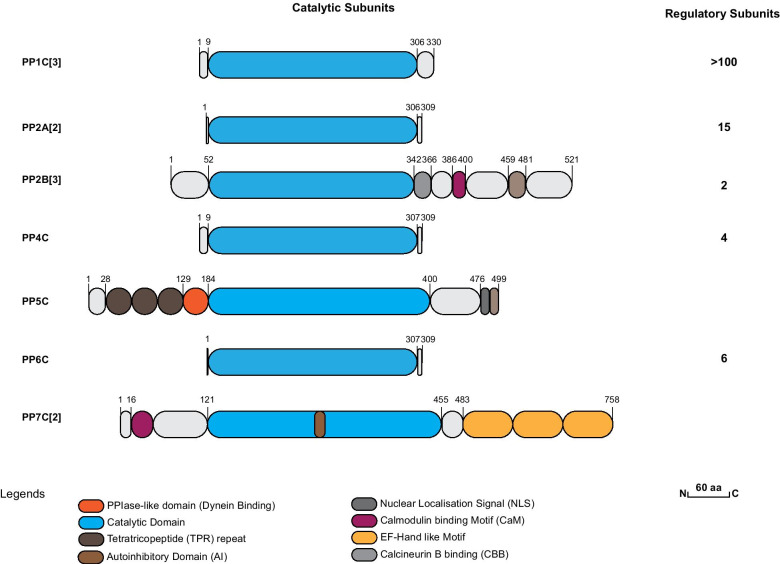
Structural organisation of members of the human PPP family. The major isoforms are depicted schematically. PPPs share high homology in the amino-terminal part of the catalytic core domain, but the carboxy-terminal parts are less similar. Autoinhibitory regions (AI), Ca^2+^-binding EF-hands, tetratricopeptide repeats (TPR), putative nuclear localisation signals (NLS), Peptidyl-Prolyl-Isomerase like domain (PPI-like), calcineurin B and calmodulin (CaM) binding sites are indicated. The number of regulatory subunits of each phosphatases is given on the right

Human PPP-family serine/threonine protein phosphatases include PP1, PP2A, PP2B (also known as calcineurin), PP4, PP5, PP6 and PP7. In general, these enzymes contain a highly conserved catalytic core domain, which can combine with a variety of regulatory subunits to determine substrate specificity and subcellular localization [40]. PP1, PP2A, PP4, and PP6 contain three characteristic sequence motifs within the central catalytic domain, differing mainly in their C- and N-terminal regions (Fig. 2). PP2B is distinct in that it contains a Ca^2+^-calmodulin (CaM) binding motif (CBD) and an autoinhibitory sequence in its C-terminal region and two divergent regions in the catalytic domain. PP5 contains in its N-terminal domain three tetratricopeptide repeats (TPR). Finally, PP7 differs from all the other phosphatases in that it contains EF-hand motifs in the C-terminal domain and a large insert within the catalytic core domain [38, 39].

## Protein phosphatase 1 (PP1)

PP1 is a ubiquitously expressed protein serine/threonine phosphatase with three homologous variants of the catalytic subunit (PP1α, PP1β, and PP1γ) encoded in the human genome. A single catalytic PP1 subunit combines with one out of nearly 200 regulatory subunits to determine substrate specificity and/or sub-cellular localization [40, 41]. In TLR-mediated innate immune responses, PP1 has been reported to play both the roles of a negative and a positive regulator.

Focusing on PP1 as a negative regulator of the TLR-mediated inflammatory response, the PP1-GADD34-CUE domain–containing 2 (CUEDC2) complex sequesters IKK-α and IKK-β in the cytoplasm and maintains them in a non-phosphorylated, inactive form. Upon stimulation with a TLR ligand (e.g. LPS), IKK is ‘recruited’ away from the PP1-GADD34-CUEDC2 module by TRAF2/TRAF6 and then is phosphorylated and activated by the TNF receptor signaling complex. Subsequently, when active IKK is released from the TNF receptor signaling complex, IKKs kinase domain binds to the carboxyl terminus of CUEDC2, presumably via TRAF2/6 [42, 43]. Via its regulatory subunit GADD34, PP1 can bind CUEDC2 and dephosphorylate IKK (IKK residues phospho-Ser-180/Ser-181), returning the kinase to its default, inactive state [44]. Interestingly, IKK does not seem to be the only kinase in the TLR-signaling cascade, which is targeted by PP1. PP1 also acts on phospho-Ser-412 of TAK-1 in macrophages and murine embryonic fibroblasts and all three catalytic subunits of PP1 (α-γ) can physically interact with TAK1 [45]. Again, PP1 mediated TAK-1 dephosphorylation depends on the regulatory subunit GADD34, which comprises a TAK-1-binding region between amino acids 242–540 neighbouring the PP1 interaction motif [45]. Phosphorylation of TAK1 residues Thr-178, Thr-184, Thr-187, and Ser-192 in the kinase activation loop is involved in maximal TAK1 activity [26], while Ser-412 is located outside of the kinase domain between the TAB1 and TAB2/3 regions of TAK1. Nevertheless, dephosphorylation of phospho-Ser-412 by PP1 appears as a key regulatory step to modulate TLR-mediated pro-inflammatory cytokine induction [45, 46]. In the context of viral infections, PP1 also negatively regulates TLR-induced IFN-α and IFN-β production. Wang and colleagues [47] identified two conserved sequences (RVLF^408^, RVFF^422^) located at the N-terminal and C-terminal ends of the DNA-binding domain of human IRF7, which match canonical PP1-binding motifs. Indeed, PP1 and IRF7 physically interact and PP1 targets four IRF7 key phosphorylation sites (S471, S472, S477, S479) [47]. Dephosphorylation of IRF7 by PP1 impairs its transcriptional activity and reduces IFN-α production upon viral infection [47]. Moreover, TLR (RLR) ligands could downregulate the kinetics of PP1 activity in macrophages. In fact, authors suggested that by means of its phosphatase activity, PP1 inhibits the full activation of IRF3 (S385, S396) leading to the decrease in TLR-mediated IFN-β expression [48].

Surprisingly, PP1γ has also been described as a positive regulator of NF-κB activation. Upon TLR-initiated MyD88 signaling, PP1γ associates with TRAF6 and promotes oligomerization of this ubiquitin E3 ligase [49]. It is suggested that PP1γ association and/or activity induce a conformational change in TRAF6 to increase its auto-ubiquitination as well as ubiquitination of the TRAF6 substrate IKKγ (NEMO), which would in the end lead to enhanced NF-κB signaling [49]. The positive contribution to NF-kB-mediated gene expression requires PP1γ phosphatase activity, but TRAF6 phospho-sites targeted by PP1γ have not been delineated [49]. Furthermore, it is unclear if a regulatory subunit of PP1γ is involved in this process. Through these events, PP1γ might contribute to physiological stimulation of NF-kB, but PP1-mediated activation of TRAF6 seems to be of particular importance in certain tumor types such as glioma cells [50] and liver cancer [51].

## Protein phosphatase 2A (PP2A)

Similar to PP1, two variants of the catalytic subunit of the serine/threonine phosphatase PP2A, termed PP2Acα and PP2Acβ, are encoded in the human genome. Either of the catalytic subunits forms the central enzyme, which combines with the scaffold subunit (PP2Aa⍺ or PP2Aaβ) and one out of 26 different regulatory subunits to build the holoenzyme complex. Again, the regulatory subunits determine the spatio-temporal specificity of PP2A enzyme activity [52–54]. A negative role of PP2A in TLR-mediated signaling was initially postulated, when application of the protein serine/threonine phosphatase inhibitor ocadaic acid resulted in increased levels of LPS-induced cytokines and chemokines [55, 56]. Additionally, reduction of PP2A expression by specific siRNAs resulted in a gain of function with regard to LPS-induced TNFα secretion further suggesting that PP2A limits TLR-induced responses [56]. Subsequently, biochemical studies have detected an interaction between PP2A and IRAK1 upon IL1β stimulation, with direct consequences for IRAK1 phosphorylation levels [34]. Potentially, there are additional targets of PP2A in the TLR signaling cascade, such as IKKα/β/γ [57], IκB-α [58], NFκB p65 [59] and MAPKs [56, 60], which have also been reported to associate with PP2A. Clear evidence for a physiological role of PP2A in TLR signaling comes from gene disruption of one of the two catalytic subunits (PP2Acα) in myeloid cells [61]. Though knock-out macrophages derived from these mice still express PP2Acβ, the total PP2A phosphatase activity was reduced by ~ 60%. This reduction was coupled to an increase in LPS-stimulated phosphorylation of proteins in the MAPK pathway (p38, JNK/c-Jun, and ERK [p44/42]) and the NF-κB pathway (IKKα/β, NF-κB p65, and IκBα) [61]. In vivo, the lack of PP2Ac⍺ in myeloid cells translated into an exaggerated inflammatory response and higher sensitivity towards LPS, with detrimental consequences for these animals upon bacterial infection [61].

One negative feed-back-loop that involves PP2A and that dampens TLR-initiated signaling is based on the lipid ceramide, which can be produced from membrane sphingolipids during inflammation [62, 63]. Ceramide either binds to PP2A directly or associates with and thereby displaces an inhibitor of PP2A termed SET [64, 65]. In both cases, PP2A phosphatase activity is unleashed by ceramide diminishing phosphorylation of multiple signaling proteins. For example during LPS-induced lung inflammation, PP2Acα is responsible for dampening cytokine release and ceramide treatment of macrophages activates PP2A to alleviate some of the acute responses and damage in the lung tissue [66]. However, despite the cumulative evidence for an important regulatory role of this protein phosphatase, the direct target(s) of PP2Ac⍺ activity and the identity of PP2Acα-associated regulatory subunits involved in the TLR signaling cascade are currently unclear.

Interestingly, PP2Ac⍺ can also associate with and de-phosphorylate serine residues in the TIR domain of MyD88 [67]. Phosphorylation of MyD88 serine residues Ser-242 and Ser-244 seems to stabilize the TLR-MyD88 complex, while PP2A-mediated dephosphorylation of these residues dampens TLR-initiated signals. This activity of PP2A is only detected during a process termed endotoxin tolerance, when immune cells have been first exposed to a low dose of LPS (0.1 µg/ml), which results in increased PP2A activity after 24 h. When these LPS-experienced cells are re-stimulated with higher doses of LPS (1 µg/ml for 15 min) the increased PP2A activity and the PP2A-mediated de-phosphorylation of MyD88 suppress TLR downstream signaling and inflammatory cytokine release [67]. Presumably, distinct PP2A holoenzymes are responsible for the different effects of this phosphatase on the initial TLR signal as described before versus the effect of PP2A on signal outcome upon TLR re-stimulation during LPS tolerance. However, it is also conceivable that differential posttranslational modification of a single PP2A holoenzyme might direct its activity towards distinct kinases in initial TLR stimulation versus the de-phosphorylation of the adapter protein MyD88 upon re-stumulation with TLR ligands.

## Protein phosphatase 2B (PP2B)/calcineurin

PP2B is a Ca^2+^/calmodulin-dependent serine/threonine phosphatase, which is also known as Calcineurin. In vertebrates, three genes (PPP3CA, PPP3CB, and PPP3CC) encode three related catalytic subunits termed Calcineurin A or PP2BC. The genes PPP3CA and PPP3CB are ubiquitously expressed, while PPP3CC transcripts are only found in testis and brain [68]. In addition, two distinct forms of the regulatory subunit, Calcineurin B, are available: Calcineurin B1, encoded by the PPP3R1 gene, is found in all cell types, while the product of the PPP3R2 gene, the protein phosphatase 3 regulatory subunit-like protein, is only found in testis [69]. The regulatory subunit Calcineurin B contains four Ca^2+^-binding EF-hand domains, while the catalytic subunit Calcineurin A contains two Calmodulin-binding sites, which are involved in regulating PP2B phosphatase activity in a Ca^2+^/Calmodulin-dependent manner [70]. Interestingly, while knock-out mice lacking either Calcineurin catalytic subunit are viable, the deletion of the regulatory subunit Calcineurin B1 leads to embryonic lethality [71]. Nevertheless, lack of either PPP3A or PPP3B impairs T cell function [72–74]. In T cells, PP2B dephosphorylates the transcription factor NFAT to promote its translocation to the nucleus, where it induces expression of cytokines such as IL-2 [75]. In addition, PP2B-mediated dephosphorylation of Bcl-10 contributes to NF-kB activity in response to TCR stimulation [76, 77]. The positive regulatory role of PP2B for T cell activation is in line with the use of PP2B inhibitors such as cyclosporin A or FK506 as immunosuppressants [68]. PP2B also has a role in the TLR-mediated innate immune response, with both negative and positive contribution to the TLR signaling pathway depending on cell type and stimulus.

For instance, PP2B acts as a negative regulator of the TLR signaling pathway in human and murine macrophages, astrocytes, and fibroblasts [78]. In macrophages, inhibition or knock-down of PP2B enhances the expression of inflammatory cytokines in response to LPS, poly(I:C), peptidoglycan, or CpG DNA, while overexpression of constitutively active Calcineurin blocks NF-κB activation by TLR ligands [79]. Protein–protein interaction and functional analysis in peritoneal macrophages revealed that PP2B interacts with TLR2, TLR4, MyD88 and TRIF, but not with TLR3 and TLR9 [79]. Accordingly, PP2B negatively regulates both MyD88-dependent and MyD88-independent TLR signaling pathways. Similar to macrophages, treatment of endothelial cells with PP2B inhibitors leads to elevated TLR4-dependent gene expression [80]. Furthermore, the treatment of kidney tubular cells with cyclosporin A or FK506 (Tacrolimus) resulted in activation of NF-κB-mediated inflammatory responses accompanied by increased TLR4/MyD88/IRAK signaling suggesting that PP2B negatively regulates this pathway [81]. In a similar manner, the application of calcineurin inhibitors led to elevated coronary arteritis in a mouse model of Kawasaki disease and this effect depended on the presence of MyD88 [82]. Though the direct target of PP2B activity in the TLR4 signaling cascade is currently unknown, overexpression of the constitutively active phosphatase reduces the phosphorylation of IRAK-1 in a dose-dependent manner [79] suggesting that PP2B acts at a position close to the activated receptor to diminish NF-kB activation. Furthermore, increased phosphorylation and degradation of IκBɑ was observed upon PP2B inhibition, suggesting a further mode of action of how this phosphatase could suppress NF-κB activity [83].

In contrast to these negative regulatory effects of PP2B on TLR signaling and NF-κB mediated gene expression events, several studies have indicated a positive contribution of PP2B to this pathway, which would be analogous to the situation in T-cell receptor signaling. For example, primary macrophages lacking a negative regulator of calcineurin-1 termed RCAN-1 show elevated inflammation in response to bacterial LPS due to enhanced TLR4-MyD88-NF-kB signaling [84]. Furthermore, PP2B inhibition by cyclosporin A selectively diminishes TLR-9-mediated IL10 expression in human B-cells [85] and treatment of mice with the PP2B inhibitor FK506 protects from LPS-induced toxicity and leads to a LPS tolerant phenotype of isolated macrophages [86]. Along the same line, peripheral blood mononuclear cells from patients receiving PP2B inhibitors show impaired inflammatory cytokine production in response to TLR2, TLR4, and TLR7/8 stimulation [87].

Together, these investigations do not draw a clear picture of PP2B’s involvement in TLR signaling. One should bear in mind that several of these studies to a large extend rely on prolonged use of Calcineurin inhibitors such as Tacrolimus or cyclophilin A. Importantly, knock-out macrophages with genetic ablation of the regulatory subunit Calcineurin B1 indicate that such pharmacological inhibitors can have effects, even when the primary target molecule is not present [82]. Therefore, contrasting reports on the role of PP2B in TLR signaling should be interpreted with caution and future emphasis should be placed on clearly delineating bone-fide protein phosphorylation sites affected by the action of PP2B/calcineurin in TLR signaling.

## Protein phosphatase 4 (PP4)

PP4 is a conserved protein serine/threonine phosphatase, which is expressed in all cell types and which is found throughout the cytoplasm and the nucleus, showing enrichment around the centrosome and microtubule nucleation points [88–90]. PP4 is composed of the PP4 catalytic subunit (PP4c), whose association with different PP4R regulatory subunits determines substrate specificity. With regard to innate immunity, PP4 has been initially reported as an activator of NF-kB-dependent transcriptional responses by dephosphorylating the c-Rel subunit of the NF-kB transcription factor [91]. Similarly, PP4 seems to act on phospho-Thr-435 of the NF-kB subunit p65 and this event correlated with increased NF-kB transcriptional activity [92]. This supposedly positive effect of PP4 on NF-kB signaling contrasts the situation with PP2A, which by dephosphorylating the RelA subunit of the NF-kB transcription factor negatively affects NF-kB transcriptional responses [59].

However, the role of PP4 in TLR signaling appears to be complex, as numerous examples of PP4′s negative regulatory function in the TLR signaling pathway became apparent. For example, the regulatory subunit R1 of PP4 (PP4R1) physically interacts with TRAF2 and TRAF6 upon LPS stimulation in murine macrophages [93, 94]. PP4 dephosphorylates specific sites in TRAF2 (Ser-11) and presumably in TRAF6, thereby blocking TRAF6 auto-ubiquitination [94]. Reduced ubiquitination of TRAF6 interferes with recruitment of TAK1 and the IKK complex ultimately reducing the activation of NF-κB [93, 94]. In line with this model, the holoenzyme PP4C-PP4R1 is proposed to regulate IKK activity and to suppress the oncogenic NF-kB signaling in T cell lymphomas. In fact, T cell activation and proliferation trigger PP4R1 expression, which is lost in cutaneous T cell lymphoma (CTCL) [95]. PP4R1 recruits PP4C to IKK and mediates the dephosphorylation of the IKK complex at residues Ser-176/Ser-180. Small interfering RNA-mediated PP4R1 depletion causes sustained and increased IKK activity and T cell hyperactivation. Furthermore, deficiency for PP4R1 in CTCL results in constitutive IKK-NF-kB signaling and thus forms an important molecular event maintaining the malignant phenotype of a subset of CTCL cells [95]. Also in the context of antiviral immunity, PP4C displays a negative regulatory function and suppresses TLR mediated production of type I IFN, as siRNA-mediated silencing of PP4C results in elevated responses towards LPS or poly(I:C) [96]. In particular, PP4C targets and dephosphorylates TBK1 at residue Ser-172. This dephosphorylation inhibits TBK1 kinase activity and thereby restrains IRF3 activation and type I IFN production [96]. Similarly, other viral agents, including polyomaviruses, exploit the PP4R1/PP4C module to interfere with NF-kB activation, thereby evading antiviral immune responses [97–99]. In these cases, the viral small T antigen connects PP4R1 and PP4C with NEMO, the scaffold of the IKK complex, and this interaction is instrumental to inhibit NF-kB activation [100]. This is reminiscent of the situation in T-cells, where PP4R1/PP4C are shown to act on IKKα and IKKβ [95] a scenario that is compatible with the findings from polyomavirus-infected cells. Together, several lines of evidence support a negative regulatory role of PP4 together with the regulatory subunit PP4R1 for NF-kB activation, while regulatory subunits of PP4 involved in promoting NF-kB signaling are currently not clearly established.

## Protein phosphatase 6 (PP6)

PP6, closely related to PP2A and PP4, is a Ser/Thr phosphatase with a bimetallic catalytic center and is expressed in most human cells and tissues [38, 101]. PP6 comprises a catalytic core domain and three regulatory subunits, namely PP6R1, PP6R2 and PP6R3, and is involved NF-kB signaling regulation. The PP6R1 is highly expressed in hematopoietic and lymphoid cells [102].

Proteomic and gene depletion analyses revealed that PP6 specifically down-regulates TAK1 activity by dephosphorylating the phospho-Thr-187 residue in the activation loop of the kinase [103]. As phosphorylation at Thr-187 is connected to increased TAK-1-induced NF-κB activity [104], it is reasonable to assume that PP6 is a negative regulator of this pathway. However, it is currently unknown, which PP6 regulatory protein is involved in this process and how the overall outcome of TLR-initited NF-κB activation is affected by PP6 activity.

## PPM family members in TLR pathway regulation

**Fig. 3:**
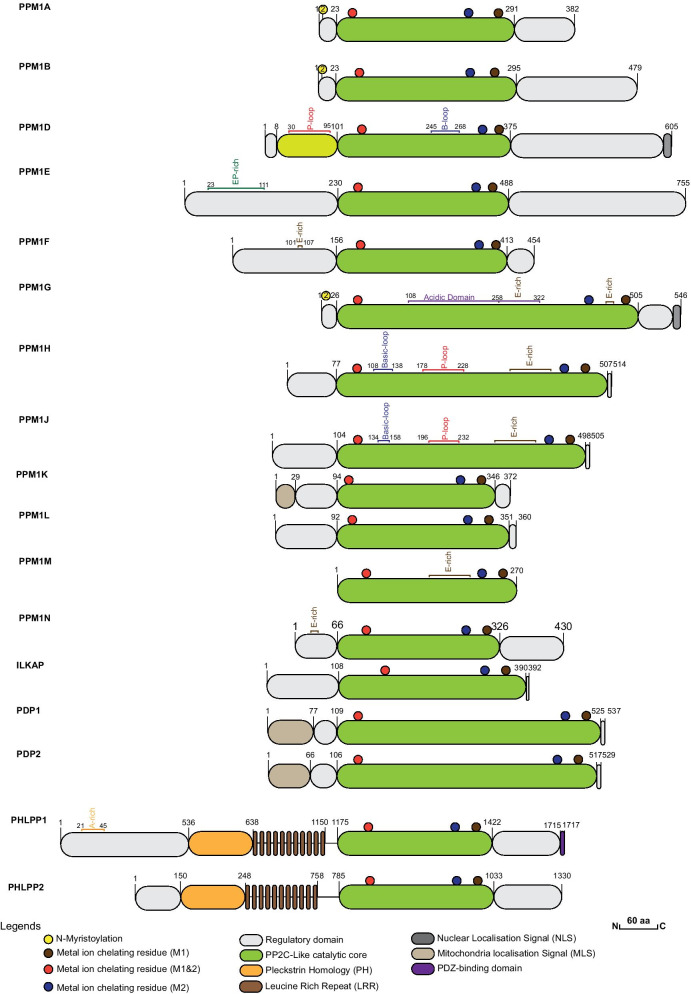
Structural organisation of members of the human PPM family. Representative members of the PPM family are depicted schematically. The PP2C-catalytic core domains of each protein are represented by the green boxes and the delimiting amino acid residues are given. The amino acids involved in chelating metal-ions are highlighted with red, blue and brown circles. N-myristoylation sites (yellow circle), sequence motifs, and protein- or lipid-binding domains are indicated

The PPM serine/threonine phosphatase family comprises 16 members (PPM1A-N) and most of them are widely expressed in human tissues [105, 106]. Structurally, these phosphatases are monomeric Mg^2+^/Mn^2+^ dependent phosphatases with a highly conserved core catalytic domain. Importantly, they generally differ from the PPP phosphatases by not forming holoenzymes. Instead, all PPM members possess N-terminal or C-terminal non-enzymatic domains, which contribute to substrate specificity and/or structural stability (Fig. 3) [38, 106–108]. Some PPM members contain, besides their phosphatase domain, typical structural folds such as a pleckstrin homology (PH) domain and a leucine-rich repeat (LRR) region in PHLPP [109, 110]. Moreover, a few PPMs contain distinct structural insertions within the catalytic core and a recent study involving PPM1G suggests that this enzyme can form a PPP-type holoenzyme using the regulatory domain B56δ of PP2A to operate in the context of adherens junction integrity [111].

Generally, PPM phosphatases are involved in diverse cellular processes such as cell proliferation, growth, survival, apoptosis, regulation of metabolism, and stress signaling [105, 106, 112–116]. However, only few studies have investigated the role of PPM phosphatases in the immune response and in particular in TLR-initiated signaling cascades.

## Protein phosphatase Mg^2+^/Mn^2+^ dependent 1A (PPM1A/α)

PPM1A is ubiquitously expressed in human tissues and, together with PPM1B, is the PPM family member, which has been studied most intensively with respect to innate immunity. [117] In the context of antiviral immunity, PPM1A was assigned an important role in governing antiviral defense and balancing anti-viral signal transduction. This phosphatase antagonizes TANK-binding kinase (TBK1)-mediated phosphorylation and aggregation of STING, which is orchestrating innate immune responses upon detection of cytosolic DNA as a sign of viral infection or mitochondrial damage [118, 119]. In addition, PPM1A antagonizes RIG-I-like receptors (RLRs), which respond to RNA viruses. Cytosolic double-stranded RNA-sensing by RLRs activates TBK1/IKKε kinases via mitochondria-associated anti-viral signaling proteins (MAVS). Analogous to counteracting TBK1 in response to DNA, PPM1A dephosphorylates the TBK1/IKKε complex and MAVS, thereby limiting the immune response against RNA viruses. As a result, PPM1A knock-out mice are resistant against RNA viruses, whereas zebrafish overexpressing PPM1A are more susceptible to RNA virus infection [120].

Likewise, PPM1A also limits TLR-initiated responses, as cytokine release upon LPS stimulation or upon *Mycobacterium tuberculosis* infection are reduced in PPM1A-overexpressing THP-1 monocytic cells [121]. In contrast, knock-down of PPM1A results in elevated levels of TNFα-release and PPM1A expression is upregulated in response to TLR stimulation by the agonists imiquimod and Pam3CSK4 [122]. These results could indicate that PPM1A operates in a negative feedback-loop to prevent an exaggerated and prolonged TLR response and to restore pre-inflammatory conditions [122].

The ability of PPM1A to dampen TLR signaling appears to hinge on the dephosphorylation of IKKβ at residues p-Ser-177 and p-Ser-181 [123]. PPM1A activity towards IKKβ terminates TNFα-induced NF-κB signaling and the same situation has been reported for PPM1B [123]. Therefore, PPM1A-mediated control of TBK1 and IKKβ phosphorylation can explain the negative regulatory role of this enzyme in TLR signaling.

## Protein phosphatase Mg^2+^/Mn^2+^ dependent 1B (PPM1B/β)

PPM1B is structurally similar to PPM1A and its genetic deletion abrogates embryonic development [124]. PPM1B plays a prominent role in inflammation by restoring the balance between apoptotic and anti-apoptotic signaling in response to TNFα in various cell lines. Together with PPM1A, PPM1B functions by specifically associating with and dephosphorylating the IKKβ complex at p-Ser-177/p-Ser-181 [125] and the upstream kinase TAK1 to terminate TNFα-induced NF-κB activity [123, 125, 126]. The adapter protein 14–3-3ε seems to be involved in the spatial and temporal co-ordination of the PPM1B—TAK1 interaction [127]. Accordingly, PPM1B is part of a negative feedback loop, which helps to limit and resolve NF-κB pathway activity at later time points following cytokine stimulation [125]. The negative feedback excerted by PPM1B on the NFκB pathway can be counter-regulated itself via PKA-mediated phosphorylation of PPM1B at Ser-195, which results in proteasomal degradation of PPM1B [128]. With regard to TLR signaling, there are striking parallels between PPM1B and PPM1A: Upon viral infection, PPM1B blocks the antiviral response by increased association with and dephosphorylation of TBK1 at Ser-172, allowing enhanced virus replication [129]. This negative regulatory function of PPM1B is exploited by certain viral proteins, which direct this phosphatase to suppress interferon production [130]. Though PPM1B has mainly been studied in the context of antiviral responses, it is highly plausible that this enzyme also controls TLR-initiated signaling during encounters with pathogenic bacteria, an area clearly demanding further exploration.

## Protein phosphatase Mg^2+^/Mn^2+^ dependent 1D (PPM1D/Wip1)

The wildtype p53-induced phosphatase 1 (Wip1, encoded by *PPM1D*) is a well-studied oncogenic member of the PPM family. It is rapidly and transiently expressed upon DNA-damaging agents and ionizing or UV irradiation in a p53-dependent manner [131–133]. As its name implies, Wip1 has a key role in restoring pre-stress cell homeostasis by controlling critical cellular functions such as proliferation, cell cycle arrest and programmed cell death after p53-dependent stress stimuli [134–138].

Wip1 is essential for immune cell development and differentiation including T and B cells, neutrophils and macrophages by regulating p53-dependent and p53-independent p38 MAPK-STAT1 pathways [138–141]. *PPM1D* knock-out mice exhibit both neutrophilia and an abnormal lymphoid histopathology in thymus and spleen, accompanied by severe defects in immune cell functions [140–142]. Not surprisingly, *PPM1D*-depleted mice also show increased susceptibility to pathogens and viral infections [143].

For example, PPM1D negatively regulates pro-inflammatory cytokine production of neutrophils after bacterial infection [144]. Accordingly, neutrophils from Wip1-deficient mice release increased amounts of pro-inflammatory cytokines and facors such as elastase, lactoferrin, and myeloperoxidase upon LPS stimulation. Apparently due to this gain in bactericidal potency, Wip1-KO mice can better contain *Staphylococcus aureus* infection in a skin abscess model [144]. On the other hand, the enhanced neutrophil functions result in pronounced LPS-induced lung damage accompanied by increased neutrophil infiltration and inflammation in the Wip1-KO mice [144]. *Ppm1d-/-*mice display a pro-inflammatory phenotype in skin and intestine with elevated levels of the cytokines TNFα, IL-6, IL-12 and IL-17 [143]. Finally, Wip1 appears to be involved in LPS-induced neuro-inflammation in a blood–brain-barrier (BBB) model, where Wip1 expression levels decreased upon LPS stimulation and the decrease in Wip1 is accompanied by augmented levels of the inflammatory cytokines TNFα, IL-1β, IL-12 and IL-6 [145].

While Wip1 expression is positively regulated by NF-κB [146], the p65 subunit of the transcription factor itself seems to be the main target of Wip1 activity [147]. In particular, Wip1 dephosphorylates p-Ser-536 of the NF-κB p65 subunit and this phospho-residue is essential for the transactivation function of p65 in recruiting the co-activator p300 [147]. Thus, this p65-directed activity of Wip1/ PPM1D can nicely explain the negative effect of this phosphatase on the expression of NF-κB-dependent inflammatory factors such as IL-1, IL-6 and IL-8 [147]. Together, the phosphatase Wip1 appears as a further PPM family member involved in negative feedback regulation of TLR-induced NF-κB signaling.

## Protein phosphatase Mg^2+/^Mn^2+^ dependent 1E (PPM1E)

The relatively large, 755 amino acids containing PPM family member PPM1E localizes to the nucleus and is predominantly expressed in brain and testis [148]. Only one prominent study on inflammatory pathway control by PPM1E has been published so far. PPM1E seems to indirectly affect TLR signaling, and its action has opposing consequences compared to other PPM family members [149]. In particular, expression of miR-135-5b in human cultured monocytes downregulates PPM1E expression and thereby activates AMPKα signaling via increased phosphorylation of AMPKa residue Thr-172. This yields in a marked attenuation of LPS-induced TNFα expression by suppression of ROS production and NF-κB activation [149]. Therefore, PPM1E positively contributes to LPS-induced responses by keeping the negative player—AMPK—under control.

## Protein phosphatase, Mg^2+/^Mn^2+^ dependent 1F (PPM1F)

The ubiquitously expressed cytoplasmic PPM1F is implicated in determining integrin-mediated cell adhesion, migration and survival and plays a key role in controlling neuronal functions by regulating Ca^2+^/Calmodulin-dependent kinase cascades [150–155]. Accordingly, *ppm1f-/-*mice show severe developmental defects and die around day E10.5 in utero [155]. PPM1F function in innate immunity has not been intensively studied, but recently PPM1F has been recognized as negative regulator of the IKK-NF-κB pathway in response to DNA-damage by dephosphorylating p-Thr-187 of TAK1 [156]. Accordingly, cells with low expression levels of PPM1F exhibit higher TAK1 activity and, in turn, show increased nuclear translocation of NF-κB and upregulation of anti-apoptotic proteins to promote cell survival and thus chemo-resistance [156]. Consequently, PPM1F might also play a role in the TLR-TAK1-NF-κB axis. Supporting this idea, Zhang et al. reported that the production of inflammatory cytokines and chemokines (IL-6, TNFα and CXCL10) was significantly augmented in THP1 PPM1F knock-down macrophages upon LPS-TLR4 stimulation [157]. Hence, it is conceivable that PPM1F suppresses the TLR4-TAK1-NF-κB signaling pathway similar to other PPM family members. Nevertheless, the molecular substrate(s) and the target residue(s) of PPM1F upon LPS stimulation still remain to be clarified.

## Protein phosphatase Mg^2+/^Mn^2+^ dependent 1L (PPM1L/ε)

PPM1L is a phosphatase localized at the endoplasmic reticulum (ER) [158]. Similar to other family members, PPM1L appears to be capable to suppress the TAK1-NF-κB pathway. Li and colleagues analyzed IL-1 / IL-1-receptor signaling and found that the activities of JNK and p38 were constantly counterregulated by PPM1L [159]. PPM1L associates with and dephosphorylates TAK1 and thereby inhibits MKK4/MKK6 binding to TAK1. On the other hand, the activity of PPM1L is diminished upon IL-1 treatment of cells resulting in transient activation of transcription factors such as AP-1 [159]. Accordingly, PPM1L appears as a further anti-inflammatory regulator [105, 159]. In line with that, a recent study assigned PPM1L a function overlapping with the activities of PPM1A and PPM1B: inhibiting NF-κB signaling via dephosphorylation of IKKβ. Clearly, PPM1L directly binds IKKβ and dephosphorylates p-Ser-177/p-Ser-181 in IKKβ, which are phosphorylated in macrophages by TAK1 upon TLR signaling [160]. Accordingly, PPM1L activity diminishes the release of inflammatory cytokines IL-1β, IL-6, TNF-α, and IL-12 [160], an activity, which could help to prevent excessive inflammatory responses.

## Protein phosphatase, Mg^2+/^Mn^2+^ dependent 1 M (PPM1M/η)

PPM1M accumulates in the nucleus and its function correlates with other members of the PPM family such as PPM1A and B in selectively acting on IKKβ downstream of TAK1 to suppress IL-1-induced NF-κB activation [161]. Accordingly, this phosphatase appears to suppress the TLR-initiated TAK1-NF-κB axis in a way similar to other PPM family members.

## Protein phosphatase Mg^2+/^Mn^2+^ dependent 1 N (PPM1N)

PPM1N is one of the least studied members of the PPM family. However, genome-wide investigations revealed that PPM1N expression in bone marrow macrophages was up-regulated upon *B. abortus* infection [162], suggesting a function of PPM1N either in the immune response towards bacteria or in inflammation.

## Conclusion

**Fig. 4:**
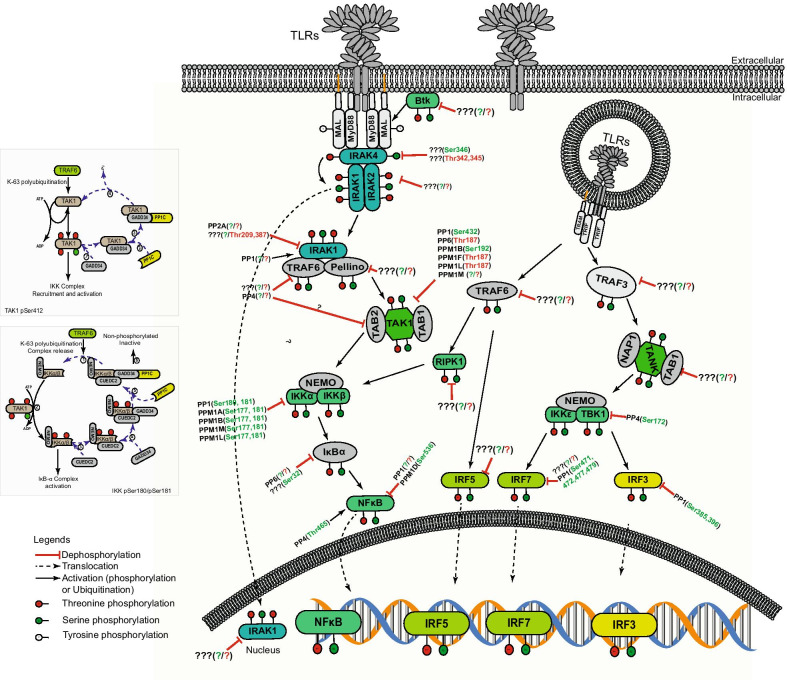
TLR-initiated signaling and the involved protein Ser/Thr phosphatases (Figs 3, 4). Summary of serine and threonine phosphorylation and de-phosphorylation events in TLR-signaling. IRAK1 phosphorylation by IRAK4 is reverted by PP2A. TRAF6 activity is inhibited by PP1 and PP4. TAK-1 has multiple phosphorylation sites, which are targeted by PP1 (Ser-432), PP6 (Thr-187), PPM1B (Ser-192), PPM1F (Thr-187), PPM1L (Thr-187), and PPM1M (unknown residues). PP1-mediated inactivation of TAK1 occurs in four sequential steps (Top frame on left). GADD34 binds to phosphorylated TAK1 (1), and recruits PP1C (2). The TAK1-GADD34-PP1C complex is formed (3) and PP1C catalyses TAK1 dephosphorylation and inactivation (4). Subsequently, the NEMO-IKK⍺/β complex phosphorylation by TAK1 is reverted by PPM1A, PPM1B, PPM1L and PPM1M on residues Ser-177 and Ser-181 and by PP1 on Ser-180/Ser-181. In the ground state, NEMO-IKKα/β complex is in a non-phosphorylated inactive form associated with GADD34/CUEDC2/PP1C (Lower frame on left). Upon stimulation, the NEMO-IKKα/β complex is released from PP1C by TRAF6 recruiment (1), IKKα and IKKβ are phosphorylated by TAK1 and subsequently trigger degradation of IκBα (2). CUEDC2 is recruited to phosphorylated IKKα/β (3), recruits GADD34 (4) and GADD34 in turn recruits PP1C (5). Finally, PP1C dephosphorylates IKKα/β and the whole complex retunrs to the ground state (6). The phosphorylation of IκBα is countered by PP6. NF-κB is de-phosphorylated by PP1, PP4 (Thr-465) and PPM1D (Ser-536). Tyrosine, serine and threonine residues are represented by white, green and red circles, respectively. Dephosphorylation is indicated by a red stop line, activation (ubiquitination or phosphorylation) and translocation are indicated by plain and dashed arrows, respectively

Identifying the involved protein phosphatases and their molecular targets in TLR signaling provides a rational basis to expand existing therapeutic strategies in acute and chronic inflammation as well as autoimmune diseases [163]. A detailed knowledge of the protein phosphatases, which counteract the various kinases downstream of TLR stimulation, could also open up novel opportunities to cope with viral or bacterial pathogens that evade host immune responses. The serine/threonine phosphatases involved in TLR signaling and their point of interception as presented in this review is summarized in  Fig. 4. It has become clear that numerous pathogenic bacteria interfere with innate immunity by exploiting host protein phosphatases. In this regard, the protein tyrosine phosphatase SHP-1 is activated by *Bordetella pertussis* to block iNOS expression and NO production in TLR-activated macrophages and also exploited by *Neisseria* gonorrhoeae or *Moraxella catarrhalis* to block IL-1β production via regulation of the SYK-TLR4-CEACAM1 complex [164, 165]. Similarly, *Listeria monocytogenes* directly hijacks PPM1A and PPM1B to dephosphorylate and translocate SIRT2 into the nucleus to promote effective infection [166]. Since these two Ser/Thr phosphatases are also an integral part of a negative regulatory feedback loop in TLR signaling, it is plausible that Listeria—triggered PPM1A and PPM1B activities also interfere with TLR-initiated innate immune signaling.

In general, pinpointing the contribution of a particular protein phosphatase to specific cellular events remains a challenge for several reasons: (i) in contrast to protein kinases there is a lack of potent and selective phosphatase inhibitors; (ii) the action of a phosphatase can in many cases only be revealed upon a positive stimulus, hampering the direct evaluation of phosphatase action; (iii) the low complexity and essential function of catalytic subunits, especially in the case of PPPs, often leads to lethal phenotypes in knock-out approaches; and (iv) redundancy between closely related enzymes, as seen for PPMs, often results in similar phenotypes upon overexpression.

The absence of highly specific enzyme inhibitors is a major obstacle for the phosphatase field in general. In the past, inhibitors such as ocadaic acid or microcystins have been widely used, but the explanatory power of such findings is often limited as PP1, PP2A, PP4, and PP6, with their extensive homology, are all sensitive to the same class of inhibitors. Thus, it is desirable, but also more demanding, to complement such pharmacologial approaches with genetic manipulation of individual phosphatases. However, one has to be aware that genetic approaches requiring prolonged selection, e.g. generation of clonal knock-out or knock-down cell lines, can trigger compensatory mechanisms that entail altered expression of related enzymes. Such compensatory activity by other, related phosphatases with overlapping substrate spectrum can easily mask a phenotype. Therefore, novel small molecule inhibitors or activators of specific phosphatases that allosterically interfere with enzyme activity of the catalytic subunit are welcome additions to the investigators toolbox [167, 168]. Furthermore, innovative strategies triggering the rapid and selective degradation of a particular phosphatase allow instant interrogation of protein function in the model system of choice [169]. Together, these approaches are poised to overcome current limitations in phosphatase research.

Given the prominent role of protein phosphorylation as a regulatory principle in TLR signaling, it is obvious, that manipulation of phosphatase activity represents a promising strategy to control innate immune responses. As we have discussed, there are numerous examples of protein phosphatases exerting negative regulatory roles in TLR signaling, but the opposite situation, a positive contribution to NF-κB activation, is also known for some phosphatase enzymes. Accordingly, a suite of small molecule stimulators as well as inhibitors of defined protein phosphatases would be ideally suited to allow a transient tuning of the TLR pathway in either direction: towards increased signaling output, when the ultimate goal is to limit infectious agents, or towards dampened responses in a situation of exaggerated or chronic inflammation. Though such approaches are still in their infancy, it is about time to unleash the translational potential of protein phosphatases in innate immunity.

## Data Availability

Not applicable.

## Abbreviations

AMPKα: 5′ AMP-activated protein kinase
AP-1: Activation protein-1
CUEDC2: Coupling of ubiquitin conjugation to ER degradation (CUE) domain-containing protein 2
DAMPs: Damage-associated molecular patterns (DAMPs)
FCP/SCP: TFIIF-associating component of RNA polymerase II C-terminal domain IL-
GADD34: Growth arrest and DNA damage-inducible protein
HAD: Halo acid dehydrogenase
1R: Interleukin 1 receptor
IL-1R: Interleukin-1 receptor
IRFs: Interferon regulatory factors
IRAKs: Interleukin-1 receptor-associated kinases
IκB: Inhibitory κappa B
IKK: IκB kinase
LPS: Lipopolysaccharides
MAPK: Mitogen-activated protein kinase
MAVS: Mitochondrial antiviral-signaling protein
MKK: Mitogen-activated protein kinase kinase
MST4: Mammalian STE20-like protein kinase 4
MyD88: Myeloid differentiation primary response 88
NEMO: NF-Kappa-B essential modulator
NFAT: Nuclear factor of activated T-cells
NF-κB: Nuclear factor kappa-light chain enhancer of activated B-cells
NIK: NF-kappa-B-inducing kinase
PAM3CSK4: Tripalmitoylated lipopeptide
PAMPs: Pathogen-associated molecular patterns
PPM: Metal-dependent protein phosphatase
PPP: Phosphoprotein phosphatases
PSPs: Protein serine/threonine phosphatases
RCAN-1: Regulator of calcineurin 1
RIPK: Receptor-interacting serine/threonine-protein kinase
RLR: Retinoic acid-inducible Gene-I-like receptor
STAT1: Signal transducer and activator of transcription 1
STING: Stimulator of interferon genes protein
TAK-1: Transforming growth factor-β-activated kinase 1
TANK: TRAF family member-associated NF-Kappa-B activator
TBK-1: TANK-binding kinase 1
TCR: T cell receptor
TIR: Toll/interleukin-1 receptor
TLR: Toll-like receptor
TRAF: Tumor necrosis factor receptor-associated factor
TRIF: TIR-domain-containing adapter-inducing interferon-β
TRAM: TRIF-related adaptor molecule
TAB1/2: TAK1-binding protein 1/2
TNF-α: Tumor necrosis factor alpha
WIP1: Wild-type p53-induced phosphatase 1
